# Does public subsidy of the cost of malaria chemoprophylaxis reduce imported malaria? A comparative policy analysis

**DOI:** 10.1186/1475-2875-12-238

**Published:** 2013-07-12

**Authors:** Penny E Neave, Steve Taylor, Ron H Behrens

**Affiliations:** 1 Department of Community Health Development, AUT University, Auckland, New Zealand; 2 Department of Biostatistics, AUT University, Auckland, New Zealand; 3 Hospital for Tropical Diseases, Mortimer Market, & London School of Hygiene and Tropical Medicine, London, UK

**Keywords:** Imported malaria, Chemoprophylaxis, Subsidized costs, Evaluation

## Abstract

**Background:**

Chemoprophylaxis is recommended for at-risk travellers visiting malaria endemic regions. The majority of travellers with imported malaria have not used this, and travellers visiting friends and relatives have the largest burden of malaria and the lowest compliance to chemoprophylaxis. In 1995, the UK’s Department of Health (DH) implemented a policy to make travellers fully responsible for the cost when purchasing chemoprophylaxis. This policy was not implemented in three Primary Care Trusts (PCTs) in London due to concern about the potential increase of imported malaria in their residents, and they maintained the public subsidy. An impact evaluation of the policy change was undertaken to determine if the continued subsidy reduced the incidence of imported malaria in one of the boroughs where the subsidy was maintained when compared to a borough where no subsidy was provided.

**Methods:**

Between 2007 and 2010 prescriptions for malaria chemoprophylaxis were collected from pharmacy records and PCTs, and all cases of imported malaria reported from the tertiary hospital in each of the two boroughs were compared.

**Results:**

The dispensed chemoprophylaxis prescriptions were nearly 8.8 times higher in Lambeth (where subsidized drugs were provided), than in Hackney. A Poisson model revealed significantly fewer reports of imported malaria per capita were made in Lambeth compared to Hackney (p = 0.042).

**Conclusions:**

The difference in malaria reports between the boroughs only just reached statistical significance, despite the considerable difference in chemoprophylaxis prescribing between the boroughs. Some travellers may not consider using chemoprophylaxis, irrespective of the cost. Regular evaluations of the recent policy changes in areas where malaria is subsidized will be important.

## Background

The use of appropriate chemoprophylaxis before travel to a malarious area is a key recommendation from the World Health Organization (WHO) for malaria prevention for at-risk travellers [1]. Atovaquone proguanil (AP), mefloquine and doxycycline are the three recommended regimens for sub-Saharan Africa and are advised by national and international bodies for all travellers to malaria endemic countries within this region. However 64% of all malaria cases reported between 1999 and 2006 were in those who acquired malaria whilst visiting friends and relatives (VFRs), and only 7% of VFRs travelling to sub-Saharan Africa took malaria chemoprophylaxis [2].

Recent research argues that AP is cost-effective compared to the cost of malaria treatment for travellers to West Africa [3]. Other estimates of the benefit of subsidized chemoprophylaxis have been made using decision tree models and data from imported cases, showing costs and benefits in travellers. Pistone *et al*. examined the likely impact of reimbursement in French residents travelling, and argued that 2,485 cases and 13 malaria deaths would have been avoided by reimbursing travellers 65% of the costs of prophylactic drugs [4]. In Switzerland, Widmer *et al*. used a similar method (decision tree model) with an enhanced sensitivity analysis on levels of subsidy and levels of compliance to prophylaxis. They calculated that if 55% of all travellers to West Africa used mefloquine, and were reimbursed 82.4% of its purchase cost, this would result in a 38% reduction of imported malaria cases with a marginal increase in costs to the health system [5].

In 1995, the UK’s Department of Health (DH) issued regulations [6] that malaria chemoprophylaxis, previously available on National Health Service (NHS) prescriptions, should no longer be subsidized [6]. The cost of unsubsidized chemoprophylaxis for an adult visiting sub-Saharan Africa for two weeks in 2009 (the middle period of the research presented here) is shown in Table 1.

**Table 1:** **Chemoprophylaxis costs and regimens for adults visiting a malarious area in sub**-**Saharan Africa**[7]

**Name of drug**	**Dosage**	**Start and length of administration**	Retail price 2009*	Cost per traveller for a 14 day visit**
Mefloquine	One tablet weekly	2-3 weeks before entering a malarious area, and four weeks after leaving	£29.06 for eight tablets	£32.69
Atavaquone plus proguanil (AP)	One tablet daily	1-2 tablets before entering a malarious area, and for one week after leaving	£50.42 for 12 tablets	£96.60
Doxycycline	One tablet daily	1-2 days before entering malarious area, and four weeks after leaving	£3.52 for 28 100mg tablets	£5.53

*retail prices are based on the profit margin suggested by the National Pharmacy Association (personal communication A Tang, NPA).

**the most common duration of travel of patients reported to the MRL is 14 days (unpublished data from the MRL 2004–2008).

In addition to the purchase costs, both General Practitioners (GPs) and community pharmacists may charge the patient for issuing and/or dispensing the private prescription. Charges vary, both for GP and community pharmacy. Following the policy change, concerns were raised by healthcare professionals that the cost of unsubsidized prescriptions may deter travellers from purchasing chemoprophylaxis for all or some family members [7] or that they may buy potentially ineffective over-the-counter drugs [8-10].

Although DH guidance is usually followed, the Health Authority in Lambeth, Southwark and Lewisham (LSL) in South London decided not to implement the 1995 DH regulations, on advice from its Public Health Department. These areas are home to relatively large numbers of people of African origin, in particular those from Nigeria and Ghana. Between 1987 and 2006, 54% of all cases of imported malaria were in those who had visited these countries [2]. The number of reports made to the Malaria Reference Laboratory (MRL) from hospitals within these boroughs is amongst the highest in the UK [2]. It was decided that chemoprophylaxis would be subsidized by the Health Authority, and it continued to be available at the cost of an NHS prescription for residents of these boroughs. This ranged from GBP 6.65 in April 2006 to GBP 7.20 in April 2010. Residents in LSL exempt from prescription charges would receive chemoprophylaxis at no cost to themselves.

Three studies carried out in different countries have investigated whether the cost of chemoprophylaxis is a deterrent to its use [11-13]. All reported that the cost of these drugs was a concern for respondents. Although this suggests that offering subsidized chemoprophylaxis may prevent malaria cases, there has to date been no evaluation of the policy of offering subsidized chemoprophylaxis in an area with a large African migrant community.

## Methods

Prescribing patterns for mefloquine and AP were compared between two boroughs administrated by different PCTs (the organisations which replaced Health Authorities and which manage the health of local populations). As doxycycline may be prescribed for reasons other than malaria chemoprophylaxis, prescriptions issued for this drug were not included.

The boroughs chosen for comparison were broadly similar with respect to key variables (Table 2) and were amongst the ten most socio-economically deprived boroughs in the country. The PCT governing Lambeth offered subsidized malaria chemoprophylaxis, whereas Hackney PCT followed the DH policy and required travellers to pay the full cost of chemoprophylaxis (Table 2).

**Table 2:** Demographic characteristics of residents in Lambeth and Hackney PCTs

	**Hackney**	**Lambeth**
Mid-year population estimate 2008 and age categorization (%)	212,200	274,500
	0-14: 44 000 (20.7)	0-14: 46 100 (16.8)
	15-64:150 100 (70.7)	15-64: 205 100 (74.7)
	65+: 18 200 (8.6)	65+: 23 100 (8.4)
Index of Multiple Deprivation ranking 2010*	2	29
Estimated number of residents of African ethnicity	21,200	23,900
Estimated number of residents born in Nigeria 2001 (%)	6,633 (3.1)	6,121 (2.2)
Estimated number of residents born in Ghana 2001 (%)	3,209 (1.5)	4,421 (1.6)

*i e Hackney was ranked the second most deprived borough in England and Lambeth was ranked the 29th from a total of 326 English boroughs [17].

It was hypothesized that there should be more prescriptions issued in Lambeth than in Hackney. The expectation was that a higher uptake of chemoprophylaxis amongst Lambeth residents would lead to a reduction in imported cases of malaria in this area.

The PCTs governing Lambeth and Hackney were asked for and provided the total number of subsidized prescriptions for AP and mefloquine issued by each GP surgery for the financial years 2007–2010. Each PCT also provided contact email addresses for each community pharmacy, which were asked to provide the numbers of unsubsidized prescriptions for AP and mefloquine dispensed between April 2007 and March 2010. Each non-responder was contacted up to three times by email and/or telephone. Counts of subsidized and unsubsidized prescriptions were combined into an Excel spreadsheet and exported into the statistical software package “R” for analysis.

The MRL provided the number of reports of malaria received from the largest hospitals situated in each borough. A Poisson model was used to test the difference in the number of reports between the boroughs, adjusting for population size.

## Results

Details were received of all subsidized prescriptions issued for AP and mefloquine (n = 28 605) for the three financial years 2007 to 2010 inclusive, for each GP practice in Lambeth (51 practices) and Hackney PCT (38 practices). Thus, the response rate was 100%.

In Hackney, 18/63 (29%) of pharmacies provided data on the number of unsubsidized prescriptions issued, whilst for Lambeth the response rate was 27/63 (43%) giving an overall response rate of prescriptions issued by pharmacies of 36%. A total of 2770 prescriptions were dispensed between 2007 to 2010 by pharmacies in these two areas. Of pharmacists who responded, 96% in Lambeth provided details of prescriptions issued in each of the three years, whilst 93% of pharmacists in Hackney gave this information (Table 3).

**Table 3:** **Prescriptions issued in Hackney and Lambeth and MRL reports** (**April 2007**-**March 2010**)

	**Hackney**			**Lambeth**		
	**Total number of subsidized prescriptions**	**Total number of unsubsidized prescriptions**	**MRL reports**	**Total number of subsidized prescriptions**	**Total number of unsubsidized prescriptions**	**MRL reports**
April 2007-March 2010	1,518	1,685	202	27,087	1,085	217
	(AP: 748, Mefloquine 770)	(AP: 688, Mefloquine: 997)		(AP:16,988,Mefloquine: 10,099)	(AP: 784, Mefloquine 301)	

There were 3,203 prescriptions issued in Hackney and 28172 issued in Lambeth over the three years (Table 3). Thus, there was an 8.8 fold increase in the number of subsidized prescriptions issued in Lambeth. In Lambeth, the more expensive AP was prescribed 70% more often than the cheaper alternative, mefloquine.

Ninety six percent of the prescriptions issued in Lambeth were subsidized, demonstrating that the majority of GPs adhered to the prescribing policy in this area. Two Lambeth GPs surgeries in particular did not follow the policy of subsidizing chemoprophylaxis, and around two thirds 398/1085 (37%) of unsubsidized prescriptions came from these. There was also some evidence that not all Hackney GPs followed the policy in operation in their area, and continued prescribing subsidized chemoprophylaxis. However, this came predominately from just three practices which issued two thirds (995/1518) of the subsidized prescriptions. This suggests that throughout other areas of Hackney, issuing unsubsidized prescriptions was the usual practice followed. All reports of laboratory-confirmed malaria (n = 419) made to the MRL for the financial years 2007 to 2010 inclusive were analysed.

The Poisson model showed that there were statistically significantly fewer reports of imported malaria per capita in Lambeth than in Hackney (p = 0.042) (Table 4).

**Table 4:** **Cumulative incidence per 100**,**000 population for prescriptions and malaria reports** (**April 2007**-**March 2010**)

	**Hackney**		**Lambeth**	
	**All prescriptions**	**MRL reports**	**All prescriptions**	**MRL reports**
	Rate per 100 000*	**Rate per 100 000**	**Rate per 100 000**	**Rate per 100 000**
April 2007-March 2010	1,521.6	96.0	10,263.0	77.9*

* p = 0.042 Poisson model.

## Discussion

This study investigated the impact of a policy of offering subsidized malaria chemoprophylaxis in one borough of London. Previous studies have argued, based on modelled data, that subsidizing malaria chemoprophylaxis would be cost effective and reduce malaria in VFR travellers [3-5]. Their findings are in contrast to this study which noted a small reduction in numbers of malaria cases when a public subsidy was provided for chemoprophylaxis. A rough estimate of the public subsidy of drug costs over this study period was GBP 1.6 million for Lambeth, whilst for Hackney the subsidized cost of AP was GBP 72,000. Even where patients with uncomplicated falciparum malaria (the most commonly acquired type of malaria reported in these areas) are managed as out-patients (as the majority are in Lambeth) the public subsidy appears to have a very marginal cost saving in reduced malaria cases.

The assumption of previous cost-benefit studies is that the cost of chemoprophylaxis determines uptake. However, other factors are likely to impact on this. For example, some VFRs might leave unused chemoprophylaxis for host friends and family in malarious countries, rather than completing their course [13]. Alternative reasons for non- adherence include a dislike of its taste or forgetfulness [13,14]. Recent research carried out to explore the reasons for the relatively high incidence of imported malaria in the Nigerian and Ghanaian communities in London suggests that not all residents who live in boroughs where malaria chemoprophylaxis is subsidized are aware of this policy [15]. Furthermore, a review of the primary research undertaken to understand risk factors for imported malaria in the African diaspora identified that as well as the cost of malaria chemoprophylaxis, low perceptions of personal risk, a distrust of non-African doctors, and problems associated with accessing drugs at short notice, for example when travelling to attend funerals were relevant [16]. Although the cost of chemoprophylaxis may be one factor deterring some individuals from purchasing it, at least some of those infected with imported malaria may not have intended to use it, irrespective of the cost. Multi-level health promotion campaigns are likely to be more effective in reducing the burden of imported malaria, rather than relying on a single cost subsidy.

There were some potential biases in the study. The numbers of reports made to the MRL from the largest hospital in each borough were compared when measuring the incidence of imported malaria. A more precise alternative would have been postcode of residence of the patient reported to the MRL but incomplete postcode data restricted its use. Travellers may be registered with GPs in boroughs different to their area of residence. Thus, some GPs may follow the policy operated in the borough in which they are located, rather than that of the area of residence of individual patients. Patients with imported malaria may be transferred to the Hospital for Tropical Diseases or other tertiary centres and so reported to the MRL from outside the borough of patient residence, but transfers were likely to be similar in both study areas.

The unsubsidized prescription data were incomplete as only one third of community pharmacists provided data, despite several reminders and there may be a potential non-response bias. Reports were received from pharmacies in all postcode areas in Hackney and all but four in Lambeth. However, the area deprivation levels in the areas of non-responders were similar to those who did respond. One of the strengths of this study was that details of all subsidized prescriptions and malaria reports were received. The study included data for three years only. A longer time frame may have yielded different findings. Under-notification of malaria cases is estimated to be around 38% in the London region [17] and may have been different between the reporting hospitals.

Cumulative incidence rates were calculated, rather than the more accurate incidence rates per person-time, and it is possible that residents in one borough may travel more frequently and for a longer duration than in the other. No local data are available on borough-level travel patterns, but there is no reason to suspect that these differ by borough of residence.

## Conclusions

This study found that subsidizing malaria chemoprophylaxis had a marginal impact on the rates of imported malaria, but was less than might be expected, given the substantial difference in the numbers of subsidized prescriptions issued by each borough. The possible biases in the study and likely alternative explanations for the findings need to be taken into account when interpreting the results, but suggest there is as yet no good evidence to support a policy for subsidizing malaria chemoprophylaxis to reduce imported malaria in London.

In October 2011 the policy in Lambeth changed, with subsidized AP no longer being prescribed unless other chemoprophylactic drugs are contraindicated [18]. Evaluations of this policy are necessary to determine their benefit, impact and cost-effectiveness.

## Abbreviations

AP: Atovaquone proguanil; DH: Department of Health; LSL: Lambeth, Southwark and Lewisham; MRL: Malaria Reference Laboratory; NHS: National Health Service; PCT: Primary Care Trust; VFR: Visiting Friends and Relatives.

## Competing interests

PEN worked as a Health Protection Specialist at the South East London Health Protection Unit. Three boroughs within South East London offered subsidized malaria chemoprophylaxis in the time period during which the study took place. ST has no competing interests. RHB is a member of the advisory board of Sigma Tau and has received research funding from Sigma Tau.

## Author’s information

PN is a Lecturer in Epidemiology and Community Environmental Health at AUT University in Auckland. She has a PhD in imported malaria in the African community in London. ST is a Biostatistician in the School of Public Health and Psychosocial Studies at AUT University in Auckland. RHB is a Senior Lecturer in the Department of Clinical Research at the London School of Hygiene and Tropical Medicine.

## Authors’ contributions

PEN jointly conceived the idea for the research, carried out the data collection, conducted some data analysis and wrote the paper. ST carried out some data analysis and commented on the possible biases in the study. RHB jointly conceived the idea for the research and contributed to writing the paper. All authors read and approved the final manuscript.
